# Synopsis and Qualitative Evaluation of a Treatment Protocol to Guide Systemic Group-Cognitive Behavioral Therapy for Misophonia

**DOI:** 10.3389/fpsyt.2022.794343

**Published:** 2022-06-28

**Authors:** Inge Jager, Nienke Vulink, Arnoud van Loon, Marthe van der Pol, Arjan Schröder, Simone Slaghekke, Damiaan Denys

**Affiliations:** ^1^Department of Psychiatry, Academic Medical Center, University of Amsterdam, Amsterdam, Netherlands; ^2^GGZ inGeest, Amsterdam, Netherlands

**Keywords:** misophonia, cognitive-behavioral therapy (CBT), protocol and guidelines, group treatment methods, psychotherapy

## Abstract

Misophonia is a disorder in which patients suffer from anger or disgust when confronted with specific sounds such as those associated with eating or breathing, causing avoidance of cue related situations resulting in significant functional impairment. Functional magnetic resonance imaging studies suggest misophonia is associated with increased activity in the auditory cortex and salience network, which might reflect increased vigilance toward specific misophonia triggers. New treatments have been developed and investigated in the last years in which this vigilance plays an important role. This is a synopsis of the first group protocol for systemic Cognitive Behavioral Therapy (G-CBT) for misophonia. We discuss the model of CBT for misophonia, provide a detailed guide to the treatment illustrated with a case study, discuss advantages, limitations, and possible pitfalls by a qualitative evaluation of the protocol, and review evidence for the protocol.

## Introduction

Misophonia is a term which has been used first in 2001 (1). It's characterization as a potential psychiatric condition was first discussed by the Denys group in 2013 (2). Research in the last two decades has been has focused primarily on its phenomenology. The diagnosis has not been added to psychiatric classification systems as DSM-V or ICD-11 yet. Misophonia is characterized by the symptoms described in Table 1, which are consistent with the symptoms observed in a group of 575 patients that allowed the revised diagnostic criteria for misophonia proposed by Jager et al. (3). Recently, a Delphi Process study (4) led to an agreement of experts on at least 80% of the consensus definition. This consensus definition corresponds highly to the Amsterdam UMC 2020 revised criteria.

**Table 1:** Revised diagnostic criteria for misophonia (3).

Amsterdam UMC 2020 revised criteria for misophonia
A. Preoccupation with a specific auditory, visual, or sensory cue, which is predominantly induced by another person. It is required that oral or nasal sounds are a trigger.
B. Cues evoke intense feelings of irritation, anger, and/or disgust of which the individual recognizes it is excessive, unreasonable, or out of proportion to the circumstances.
C. Since emotions trigger an impulsive aversive physical reaction, the individual experiences a profound sense of loss of self-control with rare but potentially aggressive outbursts.
D. The individual actively avoids situations in which triggers occur or endures triggers with intense discomfort, irritation, anger, or disgust.
E. The irritation, anger, disgust, or avoidance causes significant distress and/or significant interference in the individual's day-to-day life. For example, it is impossible to eat together, work in an open office space or live together.
F. The irritation, anger, disgust and avoidance are not better explained by another disorder, such as an Autism Spectrum Condition (e.g., a general hypersensitivity or hyper arousal to all sensory stimuli) or Attention Deficit Hyperactivity Disorder (e.g., attention problems with high distractibility in general).

Common triggers are: eating sounds (e.g., food chewing or swallowing) and nose—and breathing sounds (e.g., sniffing and heavy breathing). The intensity of the emotional response varies in different contexts and the level of stress in general. In the phenomenology of misophonia preoccupation with specific triggers is a main criterion (A). Two functional magnetic resonance imaging studies found evidence for this vigilance by showing increased activity in the auditory cortex and left amygdala in misophonia patients (5, 6). Misophonia effects quality of life; patients especially experience disabilities in family and social functioning (3, 7).

Prevalence and incidence still remain unclear, but first estimations of its prevalence suggest misophonia to be a common condition. In some reports prevalence is even estimated to be close to 20% of the population (8). In a sample of Chinese students, 6% was assessed to have misophonia (9) and in a sample of English students 12% reported moderate to severe misophonia symptoms (10). The origin of misophonia is a current topic of research. For now, we know at least a third of patients report a family history of misophonia (3, 11). Misophonia symptoms usually arise gradually in peri puberty, around the age of 13 (2, 3, 12).

Research on treatment for misophonia started in audiology with the altered intervention of tinnitus retraining therapy (TRT) (13). Currently, treatment studies exist mainly within the domain of mental health. Cognitive behavioral therapy has been investigated most often and has shown promising results in treating misophonia in single case studies (14–18).

In this article we present the first protocol for group- cognitive behavioral therapy (G-CBT) for misophonia with a systemic approach. This is a synopsis of the Dutch manual for group (G-)CBT for adults with misophonia developed by van Loon et al. (19), which contains a protocol for individual therapy and a protocol for youth (age 12–18 years) as well. The main aims of the misophonia protocol are to decrease misophonia symptoms, improve quality of life, and to provide a greater sense of personal control. The highlights of this protocol have been succinctly described in the methods section of the randomized controlled trial (20).

Even though the effect of individual CBT is still under investigation, the interventions of this manual can be applied in individual treatment as well. Because of the existing evidence for G-CBT and the significant impact of misophonia on interpersonal interactions, group therapy is very suitable for misophonia patients. In G-CBT patients find recognition among themselves and support for their symptoms. Also, in G-CBT patients have a unique chance to experience both being the victim, and offender. Knowledge of group dynamics is obviously needed to use this factor therapeutically.

The aim of the present study is to present a model for (G-)CBT for misophonia through the description of a single clinical case. This case study will serve as a running example throughout this article. We provide a detailed guide to the treatment by describing all interventions and providing timing and illustrations for the procedures. We review evidence for the protocol and will discuss advantages, limitations, and possible pitfalls by a qualitative evaluation. This article is intended as a practical guide, instead of a discussion of theoretical learning principles of CBT for misophonia.

## Methods

### Theoretical Background

Misophonia symptoms have been previously conceptualized within a cognitive-behavioral model (21–25). An extensive conceptualization within the CBT model is provided by Vollbehr and ten Broeke (2017) in the Dutch journal of cognitive and behavioral therapy, but we refer specifically to the model provided by Frank and McKay (26) and the psychological model in the recently published article of Cowan et al. (27).

**Figure 1:**
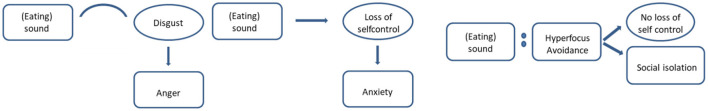
CBT conceptualization of misophonia.

Misophonia can be explained by coupling and subsequent memory consolidation of in themselves neutral stimuli (conditioned stimuli, CS) to an aversive emotional stimulus and the accompanying emotion (as a conditioned response, CR). For instance, if a child is annoyed by the sounds of his father eating, but is not allowed to leave the table and has to listen to these sounds, he or she may feel disgusted and afraid to lose control. The next meal this child will focus on the eating sounds of father again and the feelings of the last meal will come to mind. Gradually the stimulus will robustly and repeatedly evoke aversive thoughts or emotions. After this classical conditioning, principles of operant conditioning maintain and aggravate symptoms. For instance, if this child fears it cannot inhibit this intense emotional reaction to triggers it will avoid situations such as dining together, and therefore misses out on important social events. Figure 1 shows the CS-CR coupling and the avoidance and hyperfocus of the conditioned stimuli commonly seen in misophonia.

**Figure 2:**
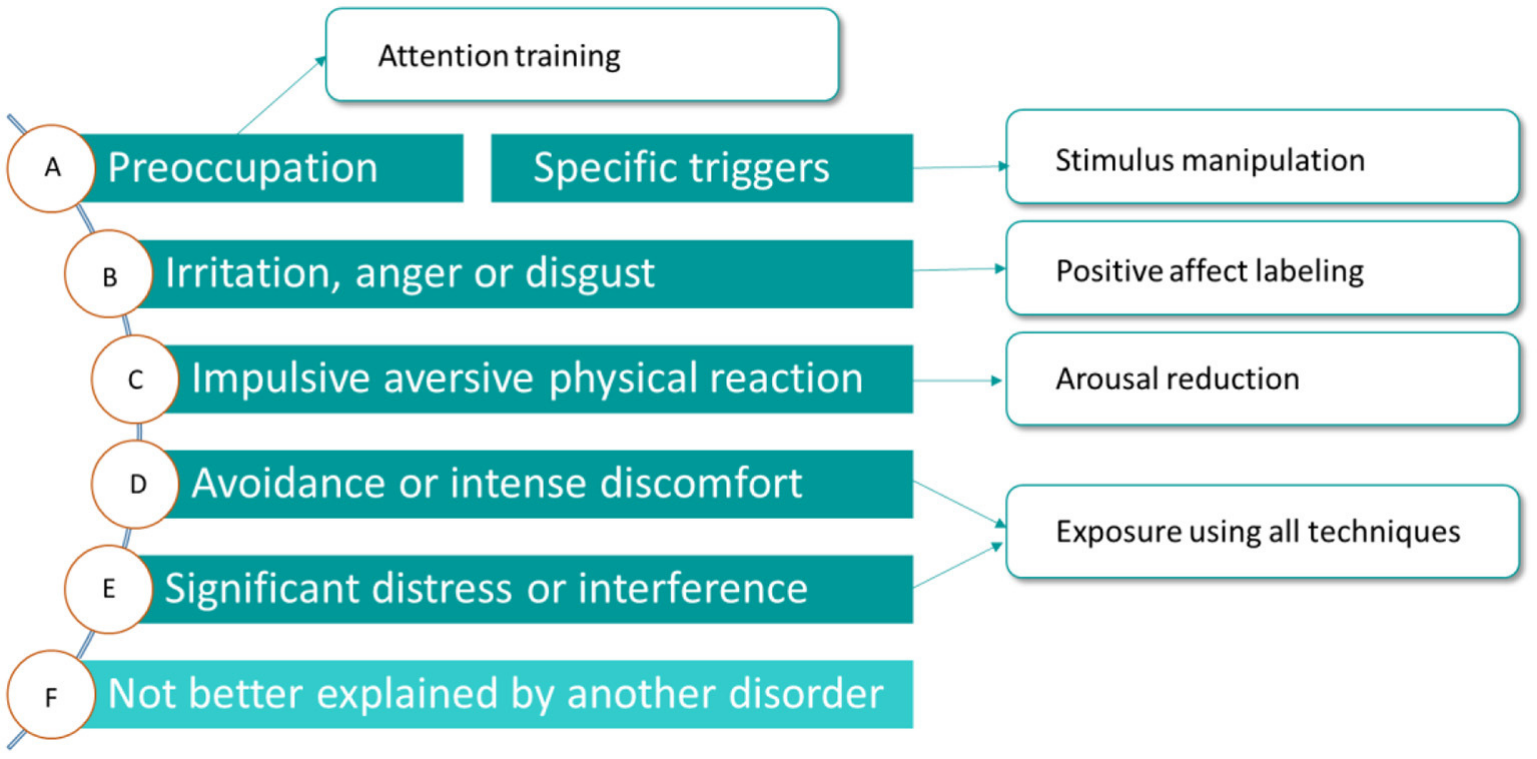
Amsterdam UMC revised criteria for misophonia and corresponding interventions.

Even though the etiology of misophonia still is unclear, the phenomenology is extensively investigated by our research group (2, 3). This research and the resulting revised diagnostic criteria for misophonia (Table 1) has been the basis of our treatment. All elements of the CBT conceptualization above can be found in the revised diagnostic criteria as well. In Figure 2, the revised diagnostic criteria on which the various interventions of the treatment manual intervene, are displayed.

The various CBT techniques intervene on different levels to maximize result. Stimulus manipulation and attention training intervene on the side of the conditioned stimulus. Arousal reduction intervenes on the conditioned response side and positive affect labeling intervenes on both the representation of the unconditioned stimulus and the conditioned response.

**Figure 3:**
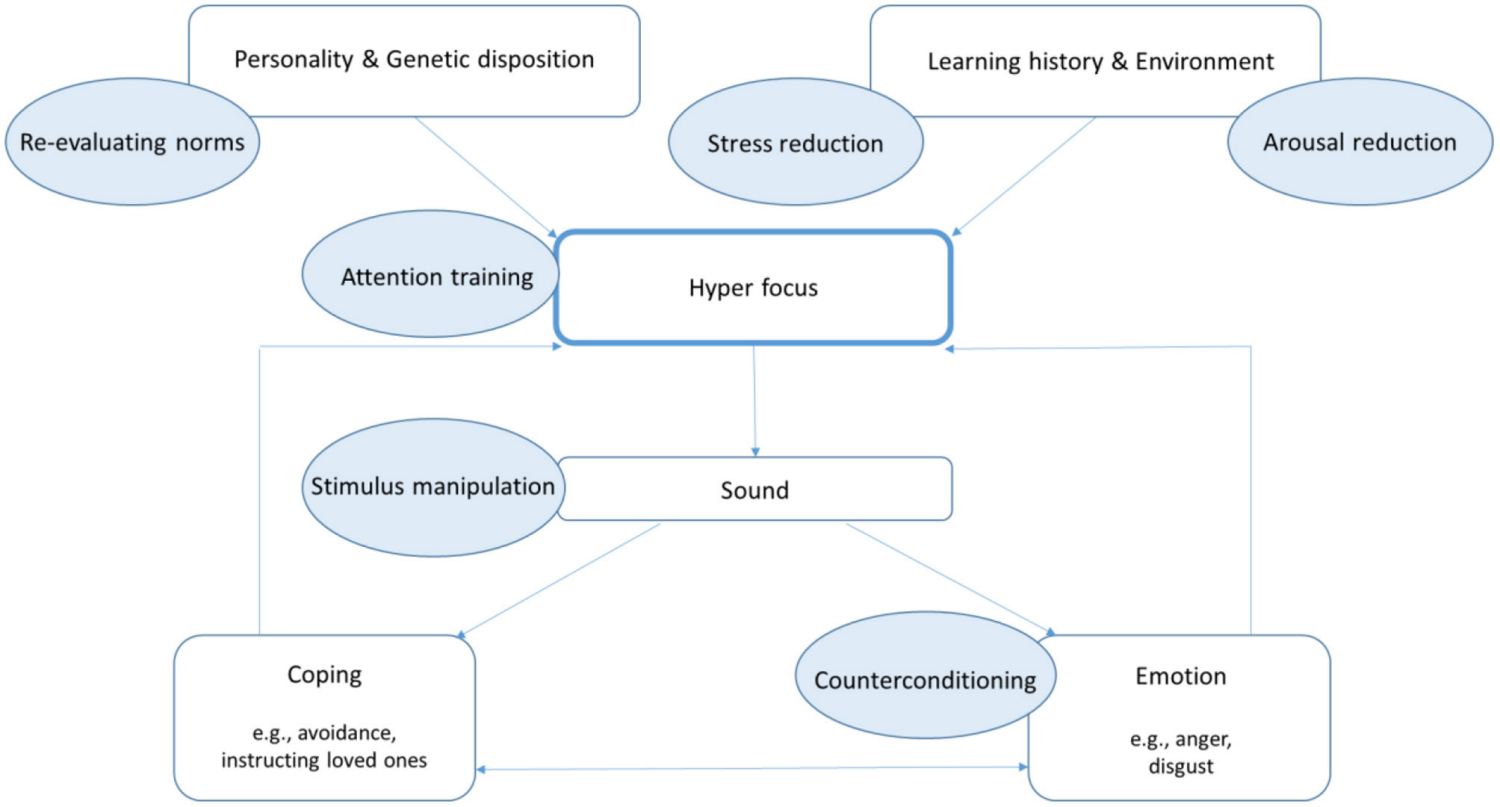
Biopsychosocial model of misophonia.

The used model for patients for characterizing misophonia in the protocol is the biopsychosocial model, presented in Figure 3.

This model assumes that *hyper focus*, characterized by preoccupation with trigger sounds and a high arousal, has a central role in the maintenance or aggravation of misophonia symptoms. Almost all patients report a hyper focus (3, 28). This hyper focus implies that patients are trained to notice trigger sounds much sooner than others. And other triggers may be added if they also are attended to easily. For example, when the sound of a spouse chewing gum is the trigger for a misophonia patient, this patient will notice this sound made by the spouse before anyone else can notice this. But it is probable this patient will then notice a colleague eating gum, and may assess if this is just as disturbing. The hyper focus then will lead to a more generalized sensitization. Besides trigger sounds, non-auditory triggers (like seeing someone chewing gum) can then cause a strong aversive emotional reaction as well. A misophonic response to visual triggers is called “misokinesia.”

Since all misophonia patients report sounds as a trigger (3, 4), it is safe to say that *sound* is the primary trigger modality in the misophonia model.

Several factors can influence the development of a hyper focus. Specific *personality* traits can include clinical perfectionism which has been found in 66–97% of patients (3), and the setting of high norms, as a trait of obsessive-compulsive personality disorder are found in 26–52% (2, 3) of patients. These traits increase the chance of developing a hyper focus. Autism like traits have not been associated with misophonia symptoms, even though the prevalence of autism spectrum conditions as a comorbid disorder in misophonia is three-fold the prevalence as in the normal population (3). Last, many of the misophonia patients report a *family history* of misophonia.

Besides factors within the patients, external factors such as learning history and environment can also contribute in the development of a hyper focus. The *learning history* of a patient with misophonia is one in which specific sounds have been associated with negative experiences by the process of classical conditioning during life. Finally, *environmental factors* influence hyper focus more directly. When patients experience stress or are tired they experience more hyper focus than when they are in good condition.

To *cope* with the trigger sounds and provoked emotions, patients develop maladaptive strategies, such as avoiding trigger sounds (e.g., they work, travel, sleep or eat alone or use earplugs), using camouflaging sounds (e.g., an extractor or music), or compulsory instructing their social environment (e.g., forbid partner to eat crisps). These strategies implemented in turn have effect on the hyper focus as depicted by the return arrow in Figure 3. For instance, a strategy such as using headphones with music, may counterintuitively lead to increased vigilance over whether the sound is still present and even greater focus on the trigger sounds despite the overlaying music. This increases hyper focus.

Trigger sounds provoke strong *emotions* of irritation and anger, and in most cases disgust (2, 12). The intensity of the aggression is mostly very strong (3). Patients quite often report the urge to harm their close relatives, because of the (eating) sounds they produce. To avoid being overwhelmed and feeling powerless by these emotions, patients pay more attention to detect trigger sounds. This is depicted by the return arrow from emotion to hyper focus in Figure 3.

### Case Conceptualization

As an illustration of the misophonia model we use the case conceptualization of an actual patient given the anonymized name of “Charlotte.” This patient meets all criteria proposed by the Amsterdam UMC in 2020. The revised Amsterdam Misophonia Scale [AMISOS-R; (29)] indicates she has severe to extreme misophonia (range 31–40). The labels in parentheses are links to the main elements of Figure 3.

Charlotte is a 37-year-old woman, who works as a lawyer and has a family with two children. Charlotte signed up for treatment, because she wants to avoid a divorce. She considers treatment as “her last straw to save her marriage.” Charlotte developed her symptoms at the age of 12, when she started puberty. She has always had high standards as a child (*personality*), but she started judging people who made more eating sounds and even disliked them. Now she considers people who make eating sounds as “people who have a defect” and refuses to interact with them. Her parents do not have misophonia, but she found out her father's mother had similar symptoms (*genetic disposition*). Charlotte grew up in a prosperous family as an only child. Her first and main trigger sound was the sound of food chewing her mother made. During her adolescence all joint meals were in a tense atmosphere, with her mother expressing she was hurt by the non-verbal aggression of Charlotte, and her father “trying to mediate between them.” Sometimes Charlotte was allowed to listen to a portable music player during dinners, but more often she was told by her father to stay at the table and control herself. This made her feel extremely powerless and she felt guilty for ruining dinner (*learning history*). During holidays, without her busy schedule of extracurricular activities, misophonia symptoms were less present. With lower stress and, as such, less sensitivity to misophonic triggers, she could enjoy her mother's company more (*environment*). Later in life she felt annoyed by nearby eating behaviors of students or colleagues, but misophonia symptoms were not disabling, because she could avoid her major triggers. When she visited her parents however, misophonia symptoms returned to levels that were present at puberty. The first 6 years of her relationship with her husband, she did not experience him as a misophonia trigger. But during the pregnancy of their firstborn child, she started to respond with disgust and aggression (*emotion*) to his eating sounds and breathing or snoring. She also had a strong reaction to the sounds of doors closing loudly, whispering, sniffing, “s” and “t” sounds, glasses being put on the table, ringing keys and the dishwasher during nighttime (*trigger sound*). She developed a strong focus on these sounds, which made it impossible to engage in social interaction or sleep during these sounds (*hyper focus*). Subsequently Charlotte avoided eating together with her family and started sleeping alone. She tried to correct her husband when he was eating, even though she realized he did not produce too much sound, and she picked many fights about his breathing sounds (*coping*).

### Phases of the Protocol

The phased structure of this G-CBT protocol for misophonia is outlined below.

(A) Assessment and engagement phase

Firstly, patients in the group are invited to get acquainted with each other, possibly with the use of introductory games (i.e., ice-breakers). Within the first session, group therapy rules concerning presence, confidentiality, and between session tasks are explained and the focus is to create a safe context for patients to share personal experiences. Therapists give psychoeducation about misophonia, validate the patients' experiences, normalize symptoms such as internal rage and emphasize similarities between the patients in the group. The therapeutic attitude is first of all validating and supporting. However, we believe that the use of humor in the group sessions from the start is an important element with positive results. Humor creates a distance toward the symptoms and helps patients to revise their high norms. It provides room to be more flexible and try out new behavior.

The biopsychosocial model for understanding misophonia is explained and filled with patients' experiences. Patients are motivated to share their memories of the onset of the misophonia symptoms (to the best of their knowledge) and the effect of misophonia on their life and life choices. Experiences are shared when patients present their “mood boards” (a personal collage consisting of images, texts, and samples of objects in a composition), an in-between-session task, with current negative associations with triggers and their desired associations.

A pretreatment measurement for misophonia symptoms can be performed at the first session with the revised Amsterdam Misophonia Scale [AMISOS-R; (29)]. We advise adding a questionnaire for general psychopathology, such as the Symptom Checklist-90 (SCL-90) (30). Further, the Sheehan Disability Scale (SDS) (31), used for other psychiatric and general medical conditions, can be applied to misophonia to determine the effect of treatment.

In the first phase consequences of misophonia symptoms on work, social life, and family life are discussed, as well as the pros and cons of being open about the diagnosis to family, friends or colleagues. Patients are encouraged to invite their close relatives to actively participate in the treatment. In this phase close relatives are invited for a separate meeting without the patients to provide psychoeducation about misophonia and the treatment, to share experiences, to manage expectations (e.g., no symptom reduction should be expected before week four) and to motivate them to participate actively and support their close relatives.

Expectations of treatment of all patients are discussed and information is provided about scientific research, as well as clinical experiences with the misophonia group protocol. Patients must be willing to devote the time needed for weekly sessions, as well as to devote energy to out-of-session work (e.g., homework). Goals are set within the first two sessions. Once goals have been identified and prioritized, they are operationalized, which involves defining the goals and all the steps that it will take to achieve them in concrete, observable/measurable cognitive or behavioral terms (SMART). Finally, patients are invited to examine their tension and attention as a first step toward reduction of arousal and stress with the body scan procedure (32).

(B) Change strategy phase

Once the secure base of the group is formed and goals are formulated in a SMART manner, various interventions to change are applied. Each group session has a theme matching the main intervention with corresponding psychoeducation and exercises (e.g., Misophonia models, Perception and attention, Stress, Conditioning, and Norms). For misokinesia psycho-education about the function of for instance wobbling legs or playing with your hair (re-evaluating norms) or the simple instruction to stop watching (attention training), is often sufficient to reduce symptoms. Additionally, fantasy and humor can be used, such as imagining a ball on a wobbly leg (counterconditioning).

Patients learn to gain control over their (internal) reactions to misophonia triggers and practice new behavior and adaptive coping strategies. The interventions are described in detail below in the Section “Overview of the Protocol.” Since patients first need to practice the various techniques before they can apply them to misophonia trigger situations, actual change often only emerges after session four. The protocol should be adapted to the different severity levels; leave patients in control when you align which steps are feasible, but always start with mild triggers.

In this stage avoidance behavior is phased out, which means patients are gradually exposed to misophonia triggers situations. The inhibitory learning model (33) is used for exposure, which emphasizes new learning when confronted with previously avoided stimuli rather than merely the cessation of fear or aversive emotional responding (26). Our systemic approach of misophonia is evidenced by the role of close relatives in this protocol. Close relatives receive psychoeducation, share experiences amongst each other, give support with between-session-tasks and patients and family or friends practice the learned techniques together.

This phase ends with a session to practice all the learned techniques together with close relatives producing trigger sounds under supervision of the therapists.

(C) Consolidation phase

The aim of the final two sessions is to develop a plan of action for the maintenance of gains and for relapse prevention. Patients practice with their misophonia triggers and exercises are done in real-life situations (e.g., visiting a food court). A list of remaining safety and avoidance behavior is made and patients make a specific plans to reduce their maladaptive misophonia behavior. This phase involves collaboration of the system. Patients and their close relatives practice together at home. For example, a spouse is “allowed” to make trigger sounds for a limited period of time, while the patient applies attention training or counterconditioning. The time of practice is gradually extended conform inhibitory learning principles.

Treatment is evaluated by discussing the effect of the different interventions, providing feedback for the therapists and a final measure of the misophonia symptom questionnaire (AMISOS-R) and possibly of general psychopathology (SCL-90) or quality-of-life (SDS) questionnaires.

### Overview of the Protocol

The protocol is designed for a closed group of maximum nine patients with seven weekly meetings and one follow-up meeting after 3 weeks. Therapy sessions last 180 min with a short break. The manual has specific instructions for each session (e.g., with a set time for each intervention and fully written exercises). CBT consists of four main components: *stimulus manipulation, counterconditioning, arousal reduction*, and *task concentration exercises*. In the most recent version two smaller elements are added: *re-evaluating (eating) norms* and *stress reduction*. Matching themes are, respectively, Perception, Conditioning, Stress, Attention, and Norms. In our center, the practical exercises, like task concentration exercises, are guided by psychomotor therapists in an exercise room, but this is not a necessity. Table 2 offers an overview of the sessions including themes, psychoeducation, in session work and homework assignments. The time of each procedure is provided in minutes. The time displayed at “Homework” refers to the explanation of the assignments. It should be noted there is a need for flexibility and the manual should be used as a guide.

**Table 2:** Overview treatment protocol per session.

	**Session 1**	**Theme: Attention**	**Time**
Assessment and engagement phase	Psychoeducation	CBT and Misophonia model	30
		Attention	10
	Work in sessions	Treatment planning	5
	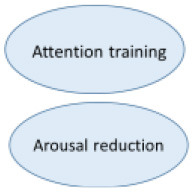	Goal setting	10
		Sharing misophonia onset *(patients read aloud their first misophonia memory)*	30
		Introduction games	40
		Attention training	45
		Bodyscan	5
	Homework	Psycho educative material *(sharing with family/ friends)*	5
		Attention training^1^	
		Applied relaxation^2^	
	**Session 2**	**Theme: Stress**	**Time**
	Psychoeducation	Stress reduction	20
		Breathing	10
	Work in sessions 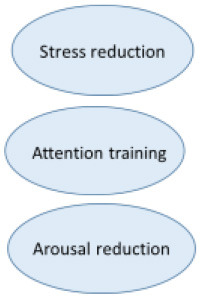	Completion misophonia models *(patients share their own model)*	45
		Attention training	45
		Applied relaxation	25
		Breathing exercises	20
	Homework	Mood boards *(one of misophonia and one of positive associations)*	15
	Stress reduction *(patients make a self-control program for stress reduction)*	
	Attention training	
	Applied relaxation and breathing exercises	
Change strategy phase	**Parallel to Session 2: Session with psychoeducation and sharing for family/friends**		90
	**Session 3**	**Theme: Perception**	**Time**
	Psychoeducation	Perception	10
	Work in sessions 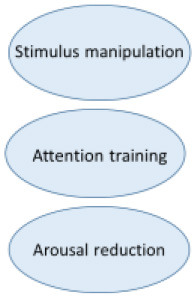	Completion stress reduction	10
		Completion mood boards *(patients present their mood boards)*	40
		Stimulus manipulation *(sound quiz and start with digital trigger sound manipulation)*	25
		Attention training	45
		Applied relaxation	30
		Breathing exercises	15
	Homework	Stimulus manipulation^3^ *(patients produce soundtracks with their triggers)*	5
		List of resembling sounds *(patients search for resembling sounds for their triggers)*	
		Attention training	
		Applied relaxation and breathing exercises	
	**Session 4**	**Theme: Conditioning**	**Time**
	Psychoeducation	Classical conditioning	25
	Work in sessions 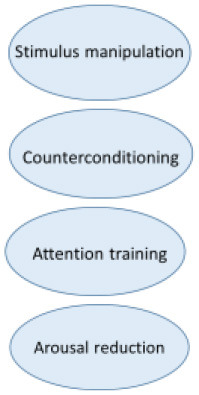	Completion stimulus manipulation *(patients present their soundtracks)*	40
		Positive affect labeling *(brainstorm counterconditioning)*	15
		Attention training	45
		Applied relaxation	30
		Breathing exercises	15
	Homework	Positive affect labeling^4^ *(patients make an audiovisual production)*	10
	Attention training	
	Applied relaxation and breathing exercises	
	**Session 5**	**Theme: Norms**	**Time**
	Psychoeducation	Misokinesia/other triggers	20
	Work in sessions 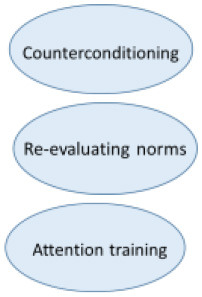	Completion positive affect labeling *(patients present their audiovisual production)*	25
		Functional analysis of (eating) norms *(panel discussion of high norms)*	40
		Attention training combined with triggers	45
		Exercises for easing high standards	40
	Homework	Positive affect labeling *(patients make new productions or extend their productions to an advertising campaign)*	10
	Behavioral experiment for high (eating) norms	
	Attention training combined with triggers	
	Applied relaxation	
	**Session 6**	**Theme: Real life**	**Time**
	Psychoeducation	–	–
	Work in sessions 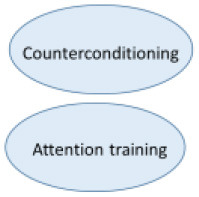	Positive affect labeling *(patients present advertising campaign or new productions)*	90
		Attention training with family/friends producing triggers	80
	Homework	Exercise plan family/friends	10
	List of misophonia behavior	
	Daily practice of the four main techniques^*^	
Consolidation phase	**Half of session 6: patients practice under guidance with family/friends**		
	**Session 7**	**Theme: Relapse prevention**	**Time**
	Psychoeducation	Relapse prevention	20
	Work in sessions 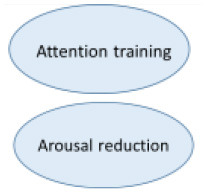	Monitoring practice	45
		Goal setting for FU	25
		Task concentration exercises and applied relaxation in public space	90
	Homework	Daily practice of the four main techniques^4^	–
	**Session 8**	**Theme: plan of action**	**Time**
	Psychoeducation	–	–
	Work in sessions	Monitoring practice	45
		Goal evaluation	20
		Goal setting for the future	25
	Homework	Daily practice of the four main techniques^1−−4^	–

In the next section, we will discuss each intervention and illustrate the interventions with the clinical case vignette of Charlotte. All interventions are displayed within Figures 2, 3.

#### Stimulus Manipulation

In stimulus manipulation the ambiguity of sounds is used as stimulus control (thus intervening on the CS). The ambiguity of sounds confuses and/or produces humor. This property of sound is illustrated by a quiz in which patients have to guess different (trigger-) sounds. Patients learn other interpretations of their trigger sounds and manipulate their trigger sounds, by editing volume or speed or merging it in different sounds or music.

For example, the sound of sniffing resembles the sound of scratching a record much like a musical DJ would do, so Charlotte mixed her colleagues' sniffing sounds into a scratched music number. When she could not see the “sniffer” but hears a sniff, she learned to imagine it was paper ripping. See for an illustration of stimulus manipulation Supplementary Video 1.

#### Counter Conditioning

The intervention of counterconditioning is used to neutralize the negative affective-evaluation of misophonia triggers (hence intervening on the UCS/UCR). This intervention shows similarities with the procedure of COMET (34). Patients produce videos where powerful personal positive images (e.g., two favorite nieces with rain boots jumping up and down in a mud puddle) are combined with the aversive misophonia trigger (e.g., food chewing) emerging with their favorite music. These videos are part of a large “campaign” with images, slogans, messages on their phone, in their house and at work, to maximize the positive affect labeling. For stimulus manipulation and counterconditioning different digital editing programs for sounds and videos can be used, for instance the free audio editing software “Audacity.” Patients who are not digitally skilled or do not have access to these programs, are encouraged to ask their close relatives for help. During therapy the group members often help each other as well. And it is almost always possible to improvise with the use of mobile phones.

Charlotte produced a video of taped breathing sounds of her spouse and edited a personal diving video of her favorite holiday with the song “A beautiful day” from the band U2. She watched the video every day, but was also reminded and counter conditioned by coral next to her bed, a picture of her wearing a diving mask as background on her phone and a quote with positive self-verbalization (“Just breathe!”) with lipstick on her mirror. See for an illustration of counterconditioning Supplementary Videos 2, 3.

#### Arousal and Stress Reduction

Arousal reduction consists of breathing techniques, progressive muscle and passive relaxation, applied relaxation techniques and mindfulness techniques (35). Initially, patients learn to reduce arousal in a normal state, later they learn to relax in a state of arousal caused by misophonic triggers. Throughout the entire treatment arousal reduction is a part of the sessions. These techniques intervene on the CR.

Stress reduction is based on an intervention for symptom reduction from the burn-out protocol (36). Patients learn through self-control techniques [e.g., (37)] to recognize symptoms of stress and manage their stress levels by either taking relaxing measures or by reducing (work-)load. Mild stress levels require small measures and higher levels require more drastic measures. Patients are made aware of the, often precarious, balance between relaxation and stress and are, if necessary, invited to make more structural changes in their work-life balance.

Charlotte noticed by registering her levels of stress that her perceived average stress level was too high. Because of her perfectionism and high standards, she often worked late after putting her children to bed. This resulted in little time to relax. She discovered the impact of stress on her misophonia; dinners on Fridays following a busy week were more difficult than on Wednesdays, when she had the afternoon off. She was convinced she had to reduce stress and made a self-control program, with signals of increasing stress (such as tension headache) and increasing measures for more relaxation (for example taking a massage or bath) or less load (such as postponing a deadline), resulting in more spare time and lower stress levels.

Whereas, breathing sounds of her spouse were a trigger for Charlotte, she learned to relax by listening to her own breathing sounds with diaphragmatic breathing. She became very skilled in applied relaxation, as she practiced this during all her breaks at her work at the law firm.

#### Attention Training

With task concentration exercises patients learn to control their attention and to shift their focus from the misophonia triggers toward the task. This technique intervenes on the CS, similar to stimulus manipulation. Difficulty of exercises gradually increases, as in the CBT protocol for social anxiety (38). First patients learn to switch their attention in situations not related to misophonia to gain experience with controlling their focus. Then, when patients are more skilled, they practice being confronted with misophonia triggers in controlled situations. Finally, they apply the attention training in real-life misophonia situations.

Charlotte first used her love for classical music to control her attention. Switching between the different instruments was easy. She practiced with shifting focus from the environment (sounds of the clock, pen clicking, or the ventilation system) to the task, for example playing badminton. At home she practiced with shifting focus from her husbands' breathing sounds to a horror movie and from eating sounds of commuters on the train to a Sudoku puzzle.

#### Re-evaluating (Eating) Norms

This intervention consists of different exercises to challenge, unconscious, assumptions and norms about eating habits or other misophonia triggers, such as sniffing. Decisional balance exercises or discussions about norms are done. Patients debate about for example the proposition: “Making eating sounds is never allowed!” This technique intervenes on the UCS/UCR.

To experience the burden of high norms and (other people's) rules, a ball game “the game without rules” is introduced. Patients can introduce new game rules, by stopping the game and putting the rule to the vote. This often results in discussion and there's no room left for playing and having fun. Finally, patients are also challenged to break their own (eating) norms in a behavioral experiment, since these norms maintain the hyper focus on triggers.

Charlotte always avoided public transport, because she detested and judged commuters who were eating in the train. She was challenged to eat a bag of her favorite crisps on the train when she was hungry. Even though she felt like a criminal at first (which amused her), she could really enjoy the crisps and could therefore slightly imagine why commuters eat while traveling.

## Results

### Development and Effectivity of the Protocol

The original manual for G-CBT was the result of years of clinical practice. Between 2011 and 2021 over 1,200 patients referred from all parts of the Netherlands with misophonia were treated within our psychiatry department. Different CBT interventions, among which cognitive therapy, exposure, and imagery rescripting, were investigated, but did not show a positive effect on the symptoms. Years of trial and error finally resulted in a mix of CBT interventions who were fine-tuned for treating misophonia in the most effective way. This protocol has been most used in the treatment of misophonia patients in clinical practice so far.

This G-CBT manual has been used in two clinical trials by our research group where it has been efficacious in treating misophonia in Dutch adults (20, 39). The effectiveness of the first version of this protocol for group treatment has been examined with good results (39). Almost half of the 90 patients studied had over 30% symptom reduction (*P* < 0.001) and were clinically assessed as “much improved” or “very much improved” on the Clinical Global Impression-Improvement (CGI-I) (40).

The protocol, with the addition of stress reduction and re-evaluating (eating) norms, has also been studied in a randomized controlled trial (RCT) in 54 patients with positive effects which were preserved at 1-year follow-up (20). In comparison with a waiting list control group treatment was effective with much to very much clinical improvement in 37% of the studied patients (*P* < 0.001) and a very large standardized effect size (d = 1.97). In all completers, on average symptoms were reduced with 28% after treatment (*P* < 0.001) and 1 year after treatment symptoms were reduced with 24% (*P* < 0.001). Thirty seven percent of the completers did not meet diagnostic criteria for misophonia any more post-treatment.

### Qualitative Evaluation of the Protocol

Treatment acceptability was quite high; 65% was (very) satisfied and 25% was neutral, and treatment was rated by patients with a mean of 6.7 out of 10 (20).

**Figure 4:**
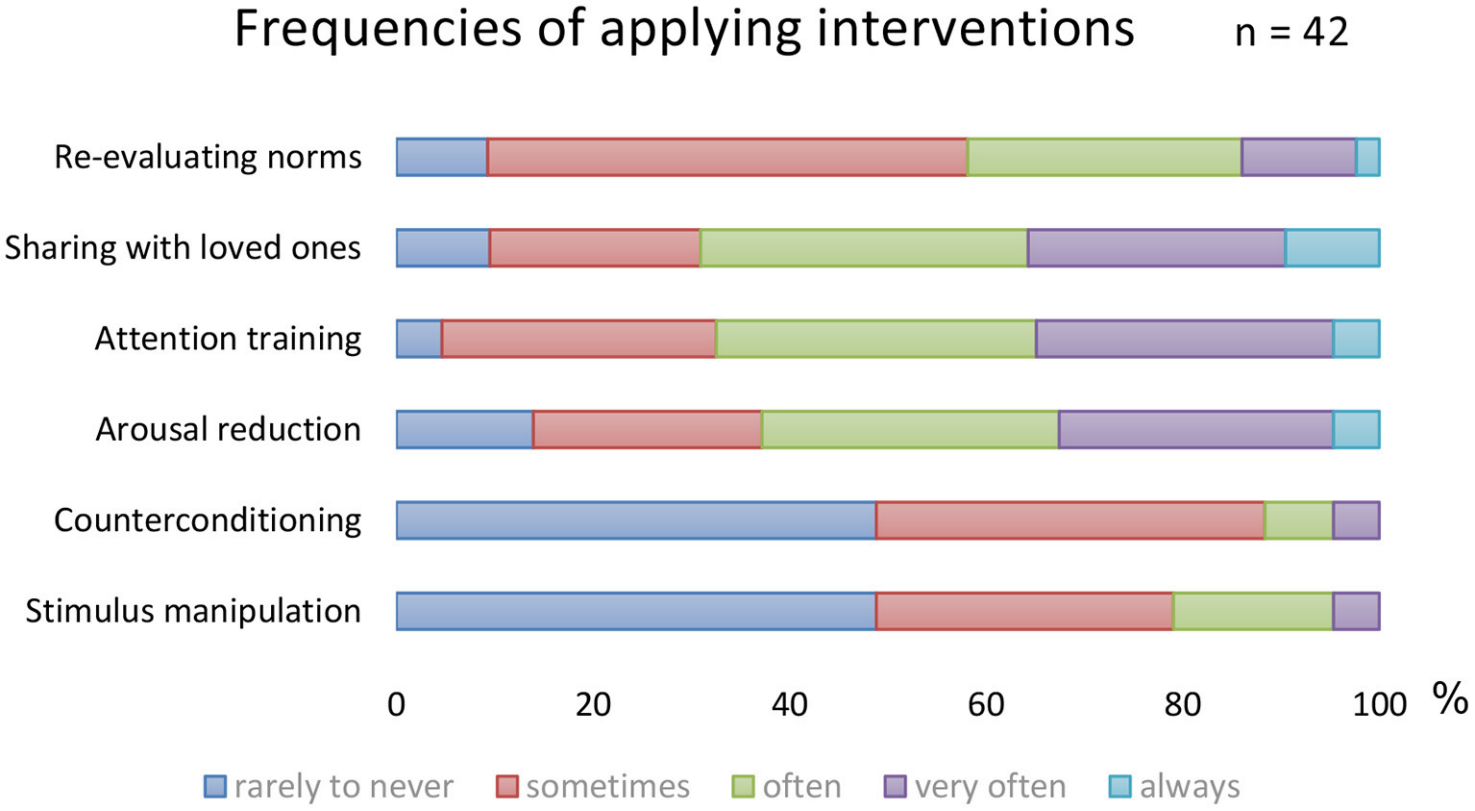
Frequencies of the CBT interventions.

The extent to which the various techniques were used and the experienced effectivity of all techniques were systematically assessed in the RCT by qualitative questionnaires post treatment (*n* = 42). Results show two of the four main interventions are applied less and are evaluated negatively. The frequencies in which the various interventions are applied are shown in Figure 4. A remarkable 48.8% “rarely to never” applies stimulus manipulation and counterconditioning. The most applied interventions are relaxation training and attention training which are used “very often” or “always” by 34.9%. Only 4.7% (attention training) to 9.3% (relaxation training) reports to “rarely to never” use these interventions.

**Figure 5:**
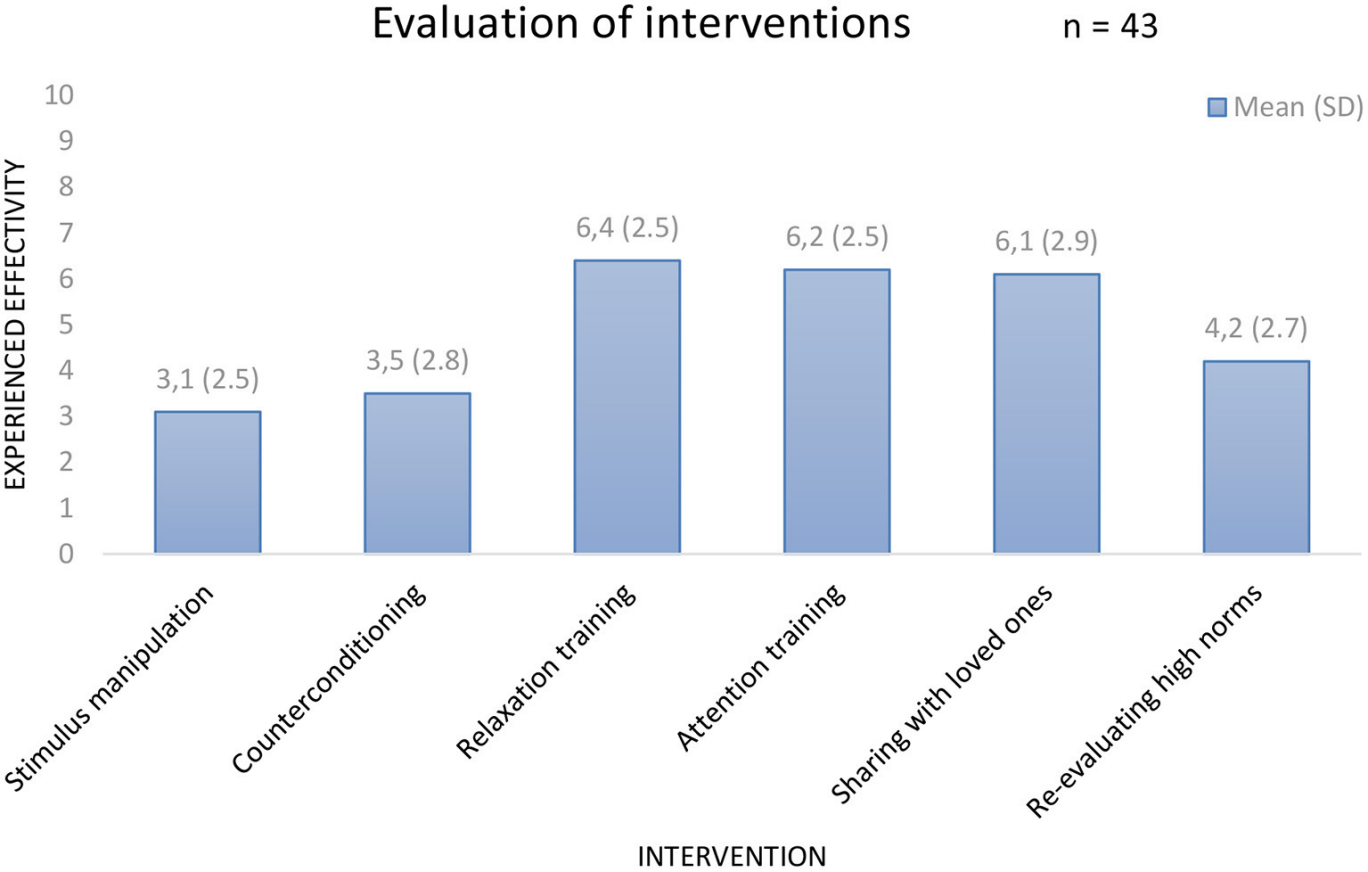
Evaluation of the CBT interventions.

In Figure 5, the results are displayed of the following question: “To what amount did the intervention contribute to your recovery?” Arousal (and stress) reduction and attention training are most highly valued. In additional comments 17 of 43 patients (40%) indicated the group element and peer support as substantially contributing to their recovery.

### Results Clinical Case Study

Results of the protocol are illustrated by the case study of Charlotte. Even though Charlotte was anxious at first to adopt new triggers from other patients, she did not. After treatment she experienced a large reduction in misophonia symptoms. Although she was still experiencing some symptoms, the relationship with her husband improved significantly. After treatment Charlotte was able to eat and sleep together again. She could make jokes with her husband about her misophonia (for example saying “Just breathe!,” when she got annoyed) and the tension at home decreased. She lost the hyper focus on most trigger sounds. The eating sounds of her mother remained a trigger for Charlotte, but she no longer avoided eating with her parents. She was able to cope in a functional manner when an emotional reaction was provoked. Charlotte stated she felt more relaxed and free in social interactions with other people.

**Figure 6:**
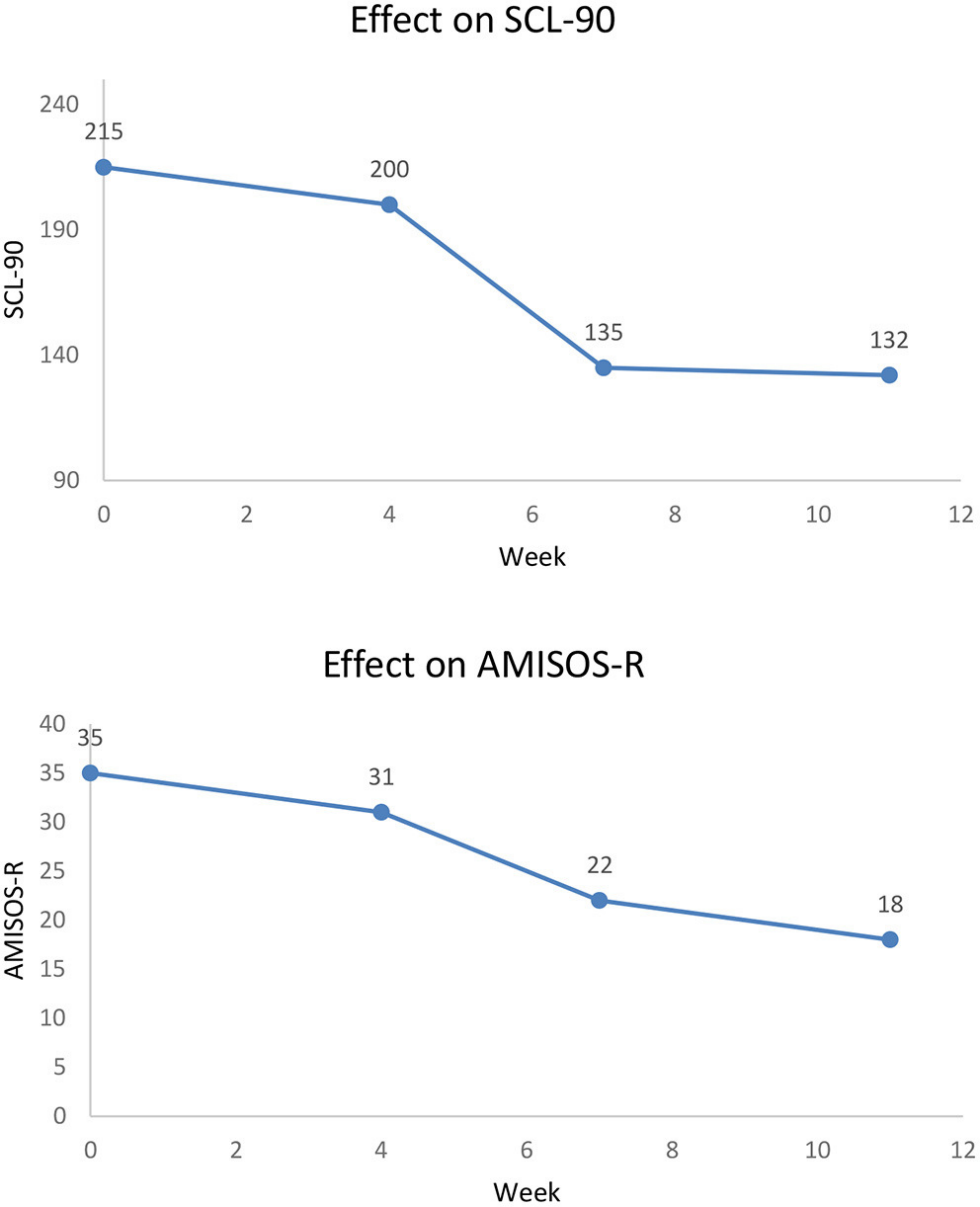
Symptoms Charlotte during G-CBT.

At session 1, 4, 7, and 8 progress was monitored by two questionnaires; the AMISOS-R and SCL-90. Charlotte started treatment with severe to extreme misophonia (range 31–40) and at the end of treatment her symptoms were reduced to mild misophonia (range 11–20). Also, general psychopathology decreased from a very high level to a level above average (see Figure 6).

## Discussion

This paper introduced the protocol for G-CBT for misophonia. The interventions are based on the revised diagnostic criteria (3). The G-CBT protocol includes various interventions: stimulus manipulation, counterconditioning, arousal and stress reduction, task concentration exercises, and re-evaluating (eating) norms. While the case study described in this paper responded to all interventions and was successfully treated by G-CBT, most patients benefit from various combinations of the interventions. More research to which elements of the treatment have most effect (on which type of patient) is needed, especially because of the lower evaluation and use of two of the main techniques. Different elements could be compared to each other or patient groups could be matched to specific interventions. For instance patients with disgust [64% according to (3)] might profit more from counterconditioning then patients without disgust. Since misophonia is an interpersonal problem with a large impact on all interactions, group therapy is very suitable. Close relatives are involved throughout the treatment, so patients are motivated to fight their misophonia together instead of fighting with each other or fighting internally.

The advantages of group treatment for misophonia consists of peer support, more opportunity to practice under guidance, more natural exposure and cost effectiveness. Compared to, for example, the case study of Muller et al. (18) with a duration of 24 sessions of 45 min of individual therapy (18 therapist-hours per patient) our treatment is brief with a duration of eight sessions of 180 min using less time per patient (6 therapist-hours per patient; in a group of eight patients with two therapists).

A first limitation of group treatment is the requirement of a number of misophonia patients. In our experience misophonia patients only admit themselves in large numbers to a center when this facility identifies itself as a misophonia treatment center. However, the described interventions can all be applied in individual treatment as well. A second limitation is the limited possibility to adjust to individual needs of patients. For example, if a patient has misophonia-related emotionally disturbing memories adding eye movement desensitization and reprocessing (EMDR) therapy as a trauma-focused approach may be considered (41). Such an additional intervention can more easily be integrated in individual therapy. A third limitation is possibly generalizability, since this protocol has been investigated in especially Dutch patient groups, with a relatively high percentage of females and Caucasians (20, 39).

The protocol has been used by one other research group so far; in a single case study by Roushani and Honarmand (42). Three patients were treated individually, but according to our protocol, with positive effect. Two of the three patients had a recovery percentage of 42–43% on anger. Further, the proposed treatment paradigm for misophonia by Frank and McKay (26) is largely based on the present protocol and includes (besides exposure using inhibitory learning): counter conditioning, stimulus manipulation and stress management in 12 sessions. Preliminary results of the 18 patients enrolled in their RCT have not been published yet. Hopefully this synopsis will contribute to the implementation of (G-)CBT in clinical trials for misophonia.

## Conclusion

In this article we have presented our treatment protocol for systemic G-CBT for misophonia, which has been evaluated in two clinical trials and has been used in clinical practice for treating over 1,200 patients. Therefore, this protocol is the worldwide most used intervention for misophonia with the highest level of evidence. In this article we have also included a qualitative evaluation of the protocol.

This protocol is based on the revised diagnostic criteria for misophonia and classical and operant learning principles. All elements are described in detail and are illustrated with a case study. It is relatively easy to adjust the group protocol to an individual approach. Knowledge of the principles of CBT, as well as a phased approach should help to maximize results.

With this treatment manual, we hope to encourage other investigators for more clinical trials and to inspire clinicians working with misophonia patients to implement (G-)CBT.

## Data Availability Statement

The raw data supporting the conclusions of this article will be made available by the authors, without undue reservation.

## Ethics Statement

Ethical review and approval was not required for the study on human participants in accordance with the local legislation and institutional requirements. The patients/participants provided their written informed consent to participate in this study. Written informed consent was obtained from the individual(s) for the publication of any potentially identifiable images or data included in this article.

## Author Contributions

IJ: conceptualization, writing—original draft, writing—review, and editing. NV and DD: conceptualization, supervision, writing—review, and editing. AL, MP, and SS: conceptualization. AS: conceptualization and writing—review. All authors contributed to the article and approved the submitted version.

## Conflict of Interest

The authors declare that the research was conducted in the absence of any commercial or financial relationships that could be construed as a potential conflict of interest.

## Publisher's Note

All claims expressed in this article are solely those of the authors and do not necessarily represent those of their affiliated organizations, or those of the publisher, the editors and the reviewers. Any product that may be evaluated in this article, or claim that may be made by its manufacturer, is not guaranteed or endorsed by the publisher.

## Supplementary Material

Supplementary Video 1 Example of stimulus manipulation: audio fragment DJ sniffing.

Supplementary Video 2 Example of counterconditioning: video fragment Baby sniffing.

Supplementary Video 3 Example of counterconditioning: video fragment Bird coughing.
